# A double shooting method for two-point boundary value problems in multibody dynamics

**DOI:** 10.1007/s11044-025-10140-7

**Published:** 2026-01-12

**Authors:** Philipp Eichmeir, Wolfgang Steiner

**Affiliations:** 1 Faculty of Engineering and Environmental Sciences, University of Applied Sciences Upper Austria, Stelzhamerstraße 23, 4600 Wels, Austria; 2 Institute of Mechanics and Mechatronics, Division of Process Control and Automation, TU Wien, Getreidemarkt 9/E325, 1060 Vienna, Austria

**Keywords:** Multibody systems, Two-point boundary value problem, Double shooting method, Multiple shooting method, Adjoint method, Optimal control

## Abstract

The solution of two-point boundary value problems (TPBVPs) in multibody system dynamics is a tough challenge. In this article, a double shooting method (DSM) tailored for TPBVPs is presented. The method builds upon the classical single shooting method while incorporating optimization strategies to overcome numerical instability and sensitivity to initial guesses. Conventional shooting methods are associated with some numerical problems: The numerical gradient computation for updating initial values causes problems in many cases, since the disturbance parameter for determining the numerical derivative must be selected appropriately in order to achieve sufficient accuracy. Moreover, integrating the initial value problem can be numerically challenging, especially when the interval is large and the differential equations have unstable modes. The DSM is designed to address these challenges. Therefore, the method incorporates a discrete costate variable approach to cope with the numerical gradient computation, which is often the Achilles’ heel of classical shooting methods. Finally, the method is formulated using a discrete implicit integration scheme to determine, as a first example, a double pendulum upswing maneuver, as a second example, the energy-optimal control of a robot multibody model and, as a third example, trajectories of the restricted three-body problem.

## Introduction

Two-point boundary value problems (TPBVPs) are of great interest for a wide range of applications in scientific and engineering disciplines, playing a major role in modeling various systems governed by ordinary differential equations (ODEs). These problems involve determining the solution to a set of ODEs subject to specified boundary conditions at two distinct points. TPBVPs occur in various fields such as heat transfer, fluid mechanics, quantum mechanics, population dynamics and optimal control theory, with applications spanning various scientific disciplines. Among the most prominent methods for solving TPBVPs are shooting methods, finite difference methods and spectral methods, which are widely used today. However, these methods are often facing challenges such as sensitivity to initial guesses, numerical instability, and computational inefficiency, particularly for large and stiff problems [12].

### Formulation of the problem of interest for this investigation

Among the various techniques for solving two-point boundary value problems, the shooting method is widely recognized and applied. The basic idea of the shooting method was originally developed by Goodman and Lance [8] more than 60 years ago and has been further developed in the subsequent years. Various authors have already discussed on the computational strategies of shooting methods [3, 19].

The single shooting method involves relatively low implementation effort due to the sequential solution of initial value problems. However, the method is associated with some numerical problems: (a) The numerical gradient computation for updating the initial value causes problems in many cases, since the disturbance parameter for determining the numerical derivative must be selected appropriately in order to achieve sufficient accuracy, (b) even small perturbations within the limits of machine precision may result in extreme deviations in the computed solution, and (c) integrating the initial value problem can be numerically challenging, especially when the interval is large and the differential equations have unstable modes. The latter issue, confer [22], is addressed by multiple shooting procedures by subdividing the interval into subintervals. A multiple shooting algorithm tailored for optimal control problems were developed in Fortran by Bulirsch [3] and by Oberle [19] at the University of Munich.

### Literature survey

Due to the long history of solving TPBVPs, a concise overview of relevant literature is provided here, highlighting both the strengths and limitations of current shooting techniques and related numerical approaches. The work by Griffith et al. [10] presents computational strategies for solving TPBVPs using an automatic differentiation tool *Object Oriented Coordinate Embedding Method*. This approach automates the computation of required partial derivatives, avoiding manual derivation and coding. Both direct and shooting methods are tested, with first-order and higher-order algorithms. In [1], a sixth-order accurate linear multi-point integration scheme for solving ODEs with two-point boundary conditions is presented. Applications include computing libration point halo orbits in restricted three- and four-body problems and designing low-thrust mission control strategies. Graichen and Petit [9] have introduced a homotopy method for TPBVPs in optimal control theory, contributing to their robustness and reliability. By employing homotopy techniques, one can transform a challenging boundary value problem into a sequence of simpler problems with known solutions, gradually deforming the problem from an initial, easily solvable form to the desired problem. This gradual deformation process helps to avoid potential singularities or numerical difficulties encountered in direct approaches. Various authors have proposed modifications of the classical shooting methods, as e.g., [6, 7, 11, 13]. More recent papers [14, 21] proposed hybrid schemes combining classical shooting algorithms with other numerical methods. While Sahu and Jena [21] integrate the shooting method with a frame operational matrix approach, in [14] the shooting method is coupled with the Secant and Newton-Raphson methods. Both approaches show an improvement in convergence compared to the classical shooting method alone. Various approaches have been proposed, including optimization-based methods, adaptive mesh refinement and hybrid schemes combining multiple techniques.

Optimization-based methods that use an adjoint gradient method to solve the underlying TPBVP of inverse dynamics and parameter identification problems are discussed in Nachbagauer et al. [17]. By coupling a standard forward solver with an adjoint backward solver, the method maintains manageable computational costs despite the complexity of the underlying equations. More recently, Pappalardo and Guida [20] present a framework for applying the adjoint method to an optimal control task of a nonlinear oscillator characterized by a generalized Van der Pol damping model. The article covers both analytical derivations and numerical algorithms, applied to feedforward and feedback control designs showing the effectiveness of the adjoint gradient method. In the work of Nejat, Moghadasi and Held [18], the adjoint gradient method is used for sensitivity analysis in multibody systems of handling kinematic loops described by differential-algebraic equations in redundant coordinates. There, the computed gradients are compared to those from a reference method using minimal coordinates.

Previous work on adaptive mesh refinement strategies can be found in the work of Bakodah et al. [2], where the solution is obtained using a hybrid approach combining the shooting method and the decomposition method. Nonetheless, there remains research potential for developing solution strategies to solve TPBVPs that balance accuracy, efficiency and robustness.

### Scope and contribution of this study

In this paper, a double shooting method (DSM) is designed to address challenges associated with shooting procedures as mentioned above and advance the state-of-the-art in TPBVP solving. Unlike traditional single shooting techniques, which are prone to numerical instability and extreme sensitivity to initial guesses, the DSM introduces a more robust framework. The challenges associated with solving boundary value problems can be considered using the Lipschitz condition, which governs how sensitive the solution is to changes in the initial conditions. The Lipschitz condition states that the rate of change of a function is bounded, meaning that small changes in the input produce proportionally small changes in the output. If a system is very sensitive to initial guesses, one can “tune” it by adjusting parameters such as using a better initial guess, employing step-size control in numerical solvers or changing the integration interval (making it smaller). Considering the latter case, a transformation of the independent variable to a unit interval can be applied, which is associated with the idea behind the multiple shooting method that the solution is more robust at smaller intervals. This transformation stabilizes the integration of potentially unstable differential equations and reduces error accumulation over long horizons. A key innovation of the method lies in the use of discrete costate variables (cf. [16]) to compute the gradient within shooting methods. By replacing conventional finite-difference approximations, which are notoriously sensitive to perturbation parameters, the DSM can evaluate gradients accurately and efficiently, addressing a long-standing bottleneck in classical shooting methods. This basic idea traces back to the adjoint variable approach [4, 5, 17, 20], which are used for optimization schemes so far. Through these features, the DSM combines the conceptual simplicity of single shooting with the robustness of multiple shooting and the computational efficiency of adjoint-based optimization, offering a new, practical tool for optimal control and dynamic simulations in complex multibody systems.

### Organization of the paper and nomenclature

The structure of the article can be summarized as follows. Section 2 introduces the formulation of the TPBVP. Section 3 presents the solution strategy of the DSM based on a discrete solver scheme. The method is formulated using a discrete scheme for an implicit integration solver and includes both the transformation to a unit interval and the costates method for the update of the initial conditions and thus offers a promising approach for solving TPBVPs. Section 4 summarizes the DSM algorithm and provides a step-by-step guide. Section 5 demonstrates the method with three numerical examples: a double-pendulum upswing maneuver, the energy-optimal control of a robotic multibody model, and trajectories in the restricted three-body problem. Finally, Section 6 concludes the article with a summary, a discussion of the advantages and limitations of the proposed approach compared to conventional boundary value problem solvers, as well as insights from previous experience and indications for future research. For improved readability, the following nomenclature summarizes the symbols used throughout this work, where bold italic letters denote vectors, bold capital letters denote matrices, and all other symbols represent scalar quantities. Parameters specific to the numerical examples are not included here to keep the list concise.

**Vectors and Matrices**

$\boldsymbol{y}(x) \in \mathbb{R}^{n}$: State vector
$\boldsymbol{u} \in \mathbb{R}^{n-l}$: Vector of unknown initial conditions
$\boldsymbol{l} \in \mathbb{R}^{l}$: Vector of prescribed left boundary conditions
$\boldsymbol{r}(\cdot ) \in \mathbb{R}^{r}$: Right boundary condition vector
$\boldsymbol{\lambda}_{i} \in \mathbb{R}^{n}$: Costate vector at time step i
$\mathbf{L} \in \mathbb{R}^{l \times n}$: Selection matrix for prescribed initial states
$\mathbf{U} \in \mathbb{R}^{(n-l) \times n}$: Selection matrix for free initial states
$\mathbf{I}_{k}$: k×k identity matrix
$\mathbf{0}_{a\times b}$: a×b zero matrix

**Functions**

$\boldsymbol{f}(\boldsymbol{y}(x),x)$: Right-hand side of the differential equations
$\boldsymbol{g}(\cdot )$: Discretized equations from the solver scheme
$\Gamma (\boldsymbol{u},a)$: Shooting error function
$\bar{\Gamma}(\boldsymbol{u},a)$: Augmented shooting error function
ℋ: Hamiltonian function
J: Performance measure (objective functional)

**Scalars**

x, $x_{0}$, $x_{f}$: Independent variable, initial and final points
$\xi \in {[0,1]}$: Normalized independent variable
$a = x_{f} - x_{0}$: Integration interval length
h: Time step size
N: Number of integration steps
η: Weighting parameter for the interval constraint

## Problem formulation

TPBVPs are constrained by boundary conditions at both ends of the domain and can be formulated by

1 $$ \begin{gathered} \boldsymbol{y}'(x) = \boldsymbol{f}(\boldsymbol{y}(x), x), \\ \boldsymbol{l} = \mathbf{L} \boldsymbol{y}(x_{0}), \quad \boldsymbol{0} = \boldsymbol{r}(\boldsymbol{y}(x_{f}), x_{f}), \end{gathered} $$

where $\boldsymbol{y}(x) \in \mathbb{R}^{n}$ is a set of functions to be determined, and $\boldsymbol{f}(\boldsymbol{y}(x),x): \mathbb{R}^{n} \times \mathbb{R} \mapsto \mathbb{R}^{n}$ represents the function defining the right-hand side of differential equations. The left boundary conditions are applied to a subset of $\boldsymbol{y}(x_{0})$ using the selection matrix $\mathbf{L} \in \mathbb{R}^{l\times n}$ by prescribing $\boldsymbol{l} \in \mathbb{R}^{l}$. Moreover, the right conditions are prescribed by $\boldsymbol{r}: \mathbb{R}^{n} \times \mathbb{R} \mapsto \mathbb{R}^{r}$, where r+l=n.

## Solution strategy

Before we apply a solution strategy to the TPBVP in Eq. (1), we transform the differential equations to a unit interval. Accordingly, a new independent coordinate, $\xi \in [0,1]$ is introduced, which is related with $x = x_{0} + a \, \xi $ where $a=x_{f}-x_{0}$. Based on the classical single shooting method, we reformulate the TPBVP from Eq. (1) as an initial value problem:

2 $$ \begin{aligned} \boldsymbol{y}'(\xi ) &= a \boldsymbol{f}(\boldsymbol{y}( \xi ), x_{0} + \xi \, a), \\ \mathbf{L} \boldsymbol{y}(0) &= \boldsymbol{l}, \\ \mathbf{U} \boldsymbol{y}(0) &= \boldsymbol{u}, \end{aligned} $$

where $\mathbf{U} \in \mathbb{R}^{(n-l) \times n}$ is a binary matrix selecting all components of $\boldsymbol{y}(0)$, which are not specified. The initial value problem in Eq. (2) can now be solved in the interval from 0 to 1 starting with the prescribed initial values $\boldsymbol{l}$ and the initial guess $\boldsymbol{u} \in \mathbb{R}^{n-l}$. If we introduce i=0,1,…,N, we can apply an implicit recursive solver scheme, e.g., an implicit Runge-Kutta method, to

3 $$ \boldsymbol{g}(\boldsymbol{y}_{i},\boldsymbol{y}_{i+1},\xi _{i},\xi _{i+1},a) = \boldsymbol{0}, $$

Solving Eq. (3) yields a sequence of function values $\boldsymbol{y}_{1},\boldsymbol{y}_{2},\dots ,\boldsymbol{y}_{N} \in \mathbb{R}^{n}$ at the integration steps $\xi _{1},\xi _{2},\dots ,\xi _{N} \in \mathbb{R}$. In order to meet the right boundary conditions in Eq. (1)_3_, we seek for initial values $\boldsymbol{u}$ and for the interval length a, minimizing the shooting error function

4 $$ \begin{aligned} \Gamma (\mathbf{u},a) = \frac{1}{2}\boldsymbol{r}^{ \mathsf {T}}(\boldsymbol{y}_{N}, x_{0}+ a) \boldsymbol{r}( \boldsymbol{y}_{N}, x_{0}+ a) + \frac{\eta}{2}\left (x_{0}-x_{f}+a \right )^{2}, \end{aligned} $$

which includes the root-mean-square error of the right boundary terms. Moreover, the length of the integration interval a is considered as an additional variable in order to stabilize the numerical solution. Therefore, we also added the root-mean-square error of the condition $0 = x_{0} -x_{f} + a$, weighted by the parameter η. Note that η=0 for a free endpoint $x_{f}$, i.e., when the interval length $x_{f} - x_{0}$ is not fixed. To compute the gradient of the shooting error function Γ with respect to $\boldsymbol{u}$ and a, Γ is augmented by the zero term from the finite difference scheme (cf. [16]) given in Eq. (3), resulting in

5 $$ \begin{aligned} \bar{\Gamma}(\mathbf{u},a) =& \, \frac{1}{2} \boldsymbol{r}^{ \mathsf {T}}(\boldsymbol{y}_{N}, x_{0}+ a) \boldsymbol{r}(\boldsymbol{y}_{N}, x_{0}+ a) + \frac{\eta}{2}\left (x_{0}-x_{f}+a \right )^{2} \\ &+ \sum _{i=0}^{N-1} \boldsymbol{\lambda}^{ \mathsf {T}}_{i} \boldsymbol{g}(\boldsymbol{y}_{i}, \boldsymbol{y}_{i+1}, \xi _{i}, \xi _{i+1}, a), \end{aligned} $$

where $\boldsymbol{\lambda}_{i} \in \mathbb{R}^{n}$ denotes a vector of costate variables. For arbitrary costate variables, the original shooting error function satisfies $\Gamma =\bar{\Gamma}$. If we introduce the abbreviations $\boldsymbol{r}_{N} := \boldsymbol{r}(\boldsymbol{y}_{N}, x_{0}+ a)$ and $\boldsymbol{g}_{i}:=\boldsymbol{g}(\boldsymbol{y}_{i}, \boldsymbol{y}_{i+1}, \xi _{i}, \xi _{i+1}, a)$, the first order variation of $\bar{\Gamma}$ in Eq. (5) is given by

6 $$ \begin{aligned} \delta \bar{\Gamma} =& \, \boldsymbol{r}_{N}^{ \mathsf {T}} \frac{\partial \boldsymbol{r}_{N}}{\partial \boldsymbol{y}_{N}} \delta \boldsymbol{y}_{N} + \left [ \boldsymbol{r}_{N}^{ \mathsf {T}} \frac{\partial \boldsymbol{r}_{N}}{\partial a} + \eta \left (x_{0}-x_{f}+a \right ) \right ] \delta a \\ &+\sum _{i=0}^{N-1} \left [ \boldsymbol{\lambda}^{ \mathsf {T}}_{i} \left ( \frac{\partial \boldsymbol{g}_{i}}{\partial a} \delta a + \frac{\partial \boldsymbol{g}_{i}}{\partial \boldsymbol{y}_{i}} \delta \boldsymbol{y}_{i} + \frac{\partial \boldsymbol{g}_{i}}{\partial \boldsymbol{y}_{i+1} } \delta \boldsymbol{y}_{i+1}\right ) \right ]. \end{aligned} $$

Performing a simple index shift of the last term of the sum in Eq. (6) yields

7 $$ \begin{aligned} \sum _{i=0}^{N-1} \boldsymbol{\lambda}^{ \mathsf {T}}_{i} \frac{\partial \boldsymbol{g}_{i} }{\partial \boldsymbol{y}_{i+1} } \delta \boldsymbol{y}_{i+1} &= \sum _{i=1}^{N} \boldsymbol{\lambda}^{ \mathsf {T}}_{i-1} \frac{\partial \boldsymbol{g}_{i-1} }{\partial \boldsymbol{y}_{i} } \delta \boldsymbol{y}_{i} = \\ &= \sum _{i=1}^{N-1} \boldsymbol{\lambda}^{ \mathsf {T}}_{i-1} \frac{\partial \boldsymbol{g}_{i-1} }{\partial \boldsymbol{y}_{i} } \delta \boldsymbol{y}_{i} + \boldsymbol{\lambda}^{ \mathsf {T}}_{N-1} \frac{\partial \boldsymbol{g}_{N-1} }{\partial \boldsymbol{y}_{N} } \delta \boldsymbol{y}_{N}. \end{aligned} $$

Thus, after substituting Eq. (7) into Eq. (6), we obtain

8 $$ \begin{aligned} \delta \bar{\Gamma} =& \, \boldsymbol{r}_{N}^{ \mathsf {T}} \frac{\partial \boldsymbol{r}_{N}}{\partial \boldsymbol{y}_{N}} \delta \boldsymbol{y}_{N} + \left [ \boldsymbol{r}_{N}^{ \mathsf {T}} \frac{\partial \boldsymbol{r}_{N}}{\partial a} + \eta \left (x_{0}-x_{f}+a \right ) \right ] \delta a + \boldsymbol{\lambda}^{ \mathsf {T}}_{0} \frac{\partial \boldsymbol{g}_{0}}{\partial a} \delta a + \boldsymbol{\lambda}^{ \mathsf {T}}_{0} \frac{\partial \boldsymbol{g}_{0}}{\partial \boldsymbol{y}_{0}} \delta \boldsymbol{y}_{0} \\ &+\sum _{i=1}^{N-1} \left [ \boldsymbol{\lambda}^{ \mathsf {T}}_{i} \frac{\partial \boldsymbol{g}_{i}}{\partial a} \delta a + \boldsymbol{\lambda}^{ \mathsf {T}}_{i} \frac{\partial \boldsymbol{g}_{i}}{\partial \boldsymbol{y}_{i}} \delta \boldsymbol{y}_{i} + \boldsymbol{\lambda}^{ \mathsf {T}}_{i-1} \frac{\partial \boldsymbol{g}_{i-1}}{\partial \boldsymbol{y}_{i} } \delta \boldsymbol{y}_{i} \right ]+ \boldsymbol{\lambda}^{ \mathsf {T}}_{N-1} \frac{\partial \boldsymbol{g}_{N-1} }{\partial \boldsymbol{y}_{N} } \delta \boldsymbol{y}_{N}. \end{aligned} $$

where an index shift has been performed for some terms in the sum. After rearranging and isolating all variations in Eq. (8), we get

9 $$ \begin{aligned} \delta \bar{\Gamma} =& \, \left (\boldsymbol{\lambda}^{ \mathsf {T}}_{N-1} \frac{\partial \boldsymbol{g}_{N-1} }{\partial \boldsymbol{y}_{N} }+ \boldsymbol{r}_{N}^{ \mathsf {T}} \frac{\partial \boldsymbol{r}_{N}}{\partial \boldsymbol{y}_{N}} \right )\delta \boldsymbol{y}_{N} \\ &+ \left [ \boldsymbol{\lambda}^{ \mathsf {T}}_{0} \frac{\partial \boldsymbol{g}_{0}}{\partial a}+ \boldsymbol{r}_{N}^{ \mathsf {T}} \frac{\partial \boldsymbol{r}_{N}}{\partial a} + \eta \left ( x_{0}-x_{f}+a \right ) \right ] \delta a \\ &+\boldsymbol{\lambda}^{ \mathsf {T}}_{0} \frac{\partial \boldsymbol{g}_{0}}{\partial \boldsymbol{y}_{0}} \delta \boldsymbol{y}_{0} + \sum _{i=1}^{N-1} \left [ \boldsymbol{\lambda}^{ \mathsf {T}}_{i} \frac{\partial \boldsymbol{g}_{i}}{\partial a} \delta a + \left ( \boldsymbol{\lambda}^{ \mathsf {T}}_{i} \frac{\partial \boldsymbol{g}_{i}}{\partial \boldsymbol{y}_{i}} + \boldsymbol{\lambda}^{ \mathsf {T}}_{i-1} \frac{\partial \boldsymbol{g}_{i-1}}{\partial \boldsymbol{y}_{i} } \right ) \delta \boldsymbol{y}_{i} \right ]. \end{aligned} $$

Imposing all variations $\delta \boldsymbol{y}_{i}$ and $\delta \boldsymbol{y}_{N}$ to vanish, i.e., by setting the coefficients to zero, we can define $\boldsymbol{\lambda}_{i}$ via a recursive scheme of the form:

10 $$ \begin{aligned} \left ( \frac{\partial \boldsymbol{g}_{N-1}}{\partial \boldsymbol{y}_{N}} \right )^{\mathsf {T}}\boldsymbol{\lambda}_{N-1} &:= -\left ( \frac{\partial \boldsymbol{r}_{N}}{\partial \boldsymbol{y}_{N}} \right )^{\mathsf {T}}\boldsymbol{r}_{N}, \\ \left ( \frac{\partial \boldsymbol{g}_{i-1}}{\partial \boldsymbol{y}_{i}} \right )^{\mathsf {T}}\boldsymbol{\lambda}_{i-1} &:= - \left ( \frac{\partial \boldsymbol{g}_{i}}{\partial \boldsymbol{y}_{i}} \right )^{\mathsf {T}}\boldsymbol{\lambda}_{i}, \end{aligned} $$

which can be solved for $\boldsymbol{\lambda}_{i}$ with i=(N−1),(N−2),…,1, starting with $\boldsymbol{\lambda}_{N-1}$. Collecting δa and $\delta \boldsymbol{y}_{0}$ in Eq. (9), the variation of the shooting error function simplifies to

11 $$ \begin{aligned} \delta \bar{\Gamma} = \boldsymbol{\lambda}^{ \mathsf {T}}_{0} \frac{\partial \boldsymbol{g}_{0}}{\partial \boldsymbol{y}_{0}} \delta \boldsymbol{y}_{0} + \left [ \boldsymbol{\lambda}^{ \mathsf {T}}_{0} \frac{\partial \boldsymbol{g}_{0}}{\partial a} + \sum _{i=1}^{N-1} \boldsymbol{\lambda}^{ \mathsf {T}}_{i} \frac{\partial \boldsymbol{g}_{i}}{\partial a} + \boldsymbol{r}_{N}^{ \mathsf {T}} \frac{\partial \boldsymbol{r}_{N}}{\partial a} + \eta \left ( x_{0}-x_{f}+a \right ) \right ] \delta a. \end{aligned} $$

By separating the variation $\boldsymbol{y}_{0}$ into constrained and free components via $\mathbf{L} \delta \boldsymbol{y}_{0} =\boldsymbol{0}$ and $\mathbf{U} \delta \boldsymbol{y}_{0}= \delta \boldsymbol{u}$, we obtain

12 $$ \begin{pmatrix} \mathbf{L} \\ \mathbf{U} \end{pmatrix} \delta \boldsymbol{y}_{0} = \begin{pmatrix} \boldsymbol{0} \\ \delta \boldsymbol{u} \end{pmatrix} , $$

or, after rearranging,

13 $$ \delta \boldsymbol{y}_{0} = \begin{pmatrix} \mathbf{L}^{ \mathsf {T}} \, \mathbf{U}^{ \mathsf {T}} \end{pmatrix} \begin{pmatrix} \boldsymbol{0} \\ \delta \boldsymbol{u} \end{pmatrix} = \mathbf{U}^{ \mathsf {T}} \delta \boldsymbol{u}. $$

Inserting Eq. (13) into Eq. (11), the update formulas for the initial conditions $\boldsymbol{u}$ and for the length of the integration interval a are identified by

14 (∂Γ¯∂ui)T=U(∂g0∂y0)Tλ0,∂Γ¯∂ai=∑i=0N−1(∂gi∂a)Tλi+(∂rN∂a)TrN+η(x0−xf+a).

Note that the binary matrix $\mathbf{U}$, which was already introduced in Eq. (2), is applied to update only the initial conditions $\boldsymbol{y}(0)$ which are not prescribed. Equation (14) can now be used for a gradient-based optimization algorithm, e.g., the Broyden-Fletcher-Goldfarb-Shanno (BFGS) method, to update previous estimates of $\boldsymbol{u}$ and a. As the functional $\bar{\Gamma}$ approaches zero, the boundary conditions of the TPBVP on the right-hand side are naturally satisfied.

## Algorithm of the DSM

We finally summarize the main steps to solve TPBVPs by the proposed DSM. In order to show how the DSM can be easily implemented, a simple collocation method is used to discretize the equations of motion. The DSM can be summarized as follows:

1. Set the initial conditions $\boldsymbol{l}$ and provide an initial guess for $\boldsymbol{u}$ and a.
2. Evaluate the left boundary conditions of the first-order differential equations by
  15 $$ \mathbf{L} \boldsymbol{y}(0) = \boldsymbol{l}, \quad \mathbf{U} \boldsymbol{y}(0) = \boldsymbol{u}. $$
3. Solve the first-order differential equations $\boldsymbol{y}' = a \boldsymbol{f}(\boldsymbol{y}(\xi ), x_{0} + \xi \, a)$ in the interval $\xi \in [0,1]$ using for example the implicit midpoint rule for i=0,1,…,N−1:
  16 $$ \boldsymbol{g}_{i} := \boldsymbol{y}_{i+1} - \boldsymbol{y}_{i} - h \, a \boldsymbol{f}\big( \bar{\boldsymbol{y}}^{+},x_{0} + a \, \bar{\xi}^{+}\big), $$
  or, respectively, for i=1,2,…,N:
  17 $$ \boldsymbol{g}_{i-1} = \boldsymbol{y}_{i} - \boldsymbol{y}_{i-1} - h \, a \boldsymbol{f}\big( \bar{\boldsymbol{y}}^{-},x_{0} + a \, \bar{\xi}^{-}\big), $$
  where
  18 $$ \begin{aligned} \bar{\xi}^{+} = \xi _{i} + \tfrac{\xi _{i+1} - \xi _{i}}{2} = \xi _{i} + \frac{h}{2}, \quad \quad \bar{\boldsymbol{y}}^{+} = \frac{\boldsymbol{y}_{i}+\boldsymbol{y}_{i+1}}{2} \phantom{.} \\ \bar{\xi}^{-} = \xi _{i-1} + \tfrac{\xi _{i} - \xi _{i-1}}{2} = \xi _{i-1} + \frac{h}{2}, \quad \quad \bar{\boldsymbol{y}}^{-} = \frac{\boldsymbol{y}_{i}+\boldsymbol{y}_{i-1}}{2}. \end{aligned} $$
  Store the solution, i.e., the function values $\boldsymbol{y}_{1},\boldsymbol{y}_{2},\dots ,\boldsymbol{y}_{N}$ and the corresponding integration steps $\xi _{1},\xi _{2},\dots ,\xi _{N}$ in the memory.
4. Compute the Jacobian matrices by
  19 ∂gi∂yii=−I−ha∂∂yif(yi+yi+12,x0+a(ξi+h2)),i=0,1,…,N−1;∂gi−1∂yi=−I−ha∂∂yif(yi+yi−12,x0+a(ξi−1+h2)),i=1,2,…,N;
  in which $\mathbf{I} \in \mathbb{R}^{n\times n}$ is an identity matrix and $\partial \boldsymbol{f}/\partial \boldsymbol{y}_{i}$ denotes partial derivatives along the forward simulation.
5. Solve the discrete costate equations from Eq. (10) by:
  20 $$ \begin{aligned} \boldsymbol{\lambda}_{N-1} &= -\left ( \frac{\partial \boldsymbol{g}_{N-1}}{\partial \boldsymbol{y}_{N}} \right )^{-\mathsf {T}} \left ( \frac{\partial \boldsymbol{r}_{N}}{\partial \boldsymbol{y}_{N}} \right )^{\mathsf {T}}\boldsymbol{r}_{N}, \\ \boldsymbol{\lambda}_{i-1} &= - \left ( \frac{\partial \boldsymbol{g}_{i-1}}{\partial \boldsymbol{y}_{i}} \right )^{-\mathsf {T}} \left ( \frac{\partial \boldsymbol{g}_{i}}{\partial \boldsymbol{y}_{i}} \right )^{\mathsf {T}}\boldsymbol{\lambda}_{i}, \end{aligned} $$
  which can be solved recursively, starting from $\boldsymbol{\lambda}_{N-1}$ and continuing backward through $\boldsymbol{\lambda}_{N-2}, \boldsymbol{\lambda}_{N-3},\dots , \boldsymbol{\lambda}_{0}$.
6. Evaluate the partial derivative $\boldsymbol{g}_{i}$ from Eq. (16) with respect to the interval length by:
  21 $$ \begin{aligned} \frac{\partial \boldsymbol{g}_{i}}{\partial a} = - h \boldsymbol{f}\left ( \tfrac{\boldsymbol{y}_{i}+\boldsymbol{y}_{i+1}}{2}, x_{0} + a \left ( \xi _{i} + \tfrac{h}{2}\right )\right ). \end{aligned} $$
7. Compute the direction of the steepest descent of the shooting error by Eq. (14).
8. Select a sufficiently small number ε>0 and update the previous estimates by $a \rightarrow a - \varepsilon ( \partial \bar{\Gamma}/\partial a)$ and $\boldsymbol{u} \rightarrow \boldsymbol{u} - \varepsilon ( \partial \bar{\Gamma}/\partial \boldsymbol{u})^{\mathsf {T}}$ as the new integration interval length and the improved initial conditions, respectively. With the updated quantities, the shooting error function should be reduced, if ε was selected sufficiently small.
9. Adjust ε, solve the first-order differential equations again and evaluate the shooting error function until an optimal value for ε is found that minimizes the shooting error function along the current search direction. Note that a line search algorithm may be used for this step.
10. Repeat steps 2 through 9 until the shooting error function approaches zero. Terminate the optimization procedure when Eq. (4) becomes sufficiently small, i.e., when the gradient is (approximately) zero.

As an alternative to steps 8–10, the gradient formula can also be passed to an optimizer, e.g., to the function fminunc from the Matlab optimization toolbox.

## Numerical examples

Three numerical examples are presented here in order to show the application of the DSM to solve TPBVPs in multibody dynamics. As a first example, an academic case of the inverse double pendulum is considered as a boundary value problem. The goal is to determine the initial velocities required to maneuver the pendulum into its upright position. As a second example, an optimal control problem of a planar robot arm is considered in order to minimize the energy of the drive torques for a point-to-point maneuver. As a third example, different trajectories of the restricted three-body problem are investigated.

### Inverse double pendulum

**Fig. 1:**
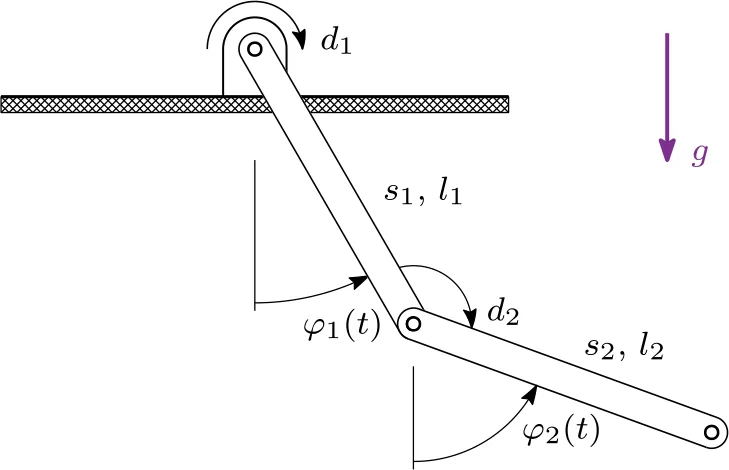
Geometric description of a double pendulum system

As a first example, an inverse double pendulum in Fig. 1 is investigated. The system is composed of two rigid bodies connected by revolute joints and has two degrees of freedom, defined by the pendulum angles $\varphi _{1}(t)$ and $\varphi _{2}(t)$. The pendulum lengths are denoted by $l_{1}$ and $l_{2}$, and the positions of their centers of gravity by $s_{1}$ and $s_{2}$. The first and second rods are characterized by the masses $m_{1}$ and $m_{2}$ and moments of inertia $J_{1}$ and $J_{2}$, respectively. In the hinge joints, a linear damping force is applied, with the damping constants $d_{1}$ and $d_{2}$. Introducing the abbreviations

22 $$ \begin{gathered} I_{a} = J_{1} + s_{1}^{2} m_{1} + l_{1}^{2} m_{2}, \quad I_{b} = J_{2} + s_{2}^{2} m_{2}, \\ I_{c} = l_{1} s_{2} m_{2}, \end{gathered} $$

and

23 $$ \begin{aligned} G_{a} = g \left ( s_{1} m_{1} + l_{1} m_{2} \right ), \quad G_{b} = g s_{2} m_{2}, \end{aligned} $$

the double pendulum system is governed by the first-order differential equations

24 $$ \begin{aligned} \dot{\varphi}_{1} =& \, \omega _{1} \\ \dot{\varphi}_{2} =& \, \omega _{2} \\ \dot{\omega}_{1} =& \, \mu \Big[ I_{b} \big( d_{1} \omega _{1} + d_{2}( \omega _{1}-\omega _{2}) + G_{a} \sin (\varphi _{1})\big) + I_{c} \big( I_{b} \omega _{2}^{2} \sin (\varphi _{1} - \varphi _{2}) \\ &+I_{c} \omega _{1}^{2} \cos (\varphi _{2}) \big) -I_{c} \big(d_{2} ( \omega _{2}-\omega _{1}) + G_{b} \big) \cos (\varphi _{1} - \varphi _{2}) \Big] \\ \dot{\omega}_{2} =& \, \mu \Big[ I_{a} \big(d_{2}(\omega _{1}-\omega _{2}) - G_{b} \sin (\varphi _{2}) \big) +I_{c} \big( I_{a} \omega _{1}^{2} + I_{c} \omega _{2}^{2} \cos (\varphi _{1}-\varphi _{2}) \big) \sin ( \varphi _{1}-\varphi _{2}) \\ &+ I_{c} \big(d_{1} \omega _{1} + d_{2} (\omega _{1}-\omega _{2}) + G_{a} \sin (\varphi _{1})\big) \cos (\varphi _{1}-\varphi _{2}) \Big], \end{aligned} $$

where the abbreviation $\mu = 1/\left (I_{a} I_{b} - I_{c}^{2} \cos ^{2}(\varphi _{1} - \varphi _{2})\right )$ is used. We consider a double pendulum that is initially positioned in its downward configuration. Now the goal is to determine the initial angular velocities that maneuvers the pendulum into the unstable upper equilibrium. This problem can be formulated as TPBVP whose solution yields the necessary conditions for the swing-up maneuver. For $t_{0}=0$ the initial conditions (left-hand side) read

25 $$ \varphi _{1}(0) = 0 \text{ rad}, \quad \varphi _{2}(0) = 0 \text{ rad}, \quad \omega _{1}(0) = \mathrm{free}, \quad \omega _{2}(0) = \mathrm{free}. $$

Moreover, the right boundary conditions ($x_{f} = T$) are prescribed by

26 $$ \varphi _{1}(T) = \pi {\text{ rad}}, \quad \varphi _{2}(T) = \pi {\text{ rad}}, \quad \omega _{1}(T) = 0~{\mathrm{rad}/s}, \quad \omega _{2}(T) = \mathrm{free} \quad T = \mathrm{free}. $$

Considering time as an additional variable, the system becomes five-dimensional. Hence, we can prescribe five boundary conditions, with $\omega _{2}(T)$ considered free to prevent the system from becoming overdetermined. When a boundary value problem is overdetermined, i.e., when the number of boundary conditions are imposed exceeds the number of differential equations, the DSM cannot satisfy all conditions exactly. In such cases, the method converges to a local minimum of the shooting error function, which is generally nonzero and does not correspond to a minimum-norm solution. It is therefore necessary to avoid overdetermination, as is also required when using other numerical solvers for boundary value problems.

#### Solution strategy

However, owing to the free final time T, a total of five boundary conditions are imposed by Eq. (25) and Eq. (26). Introducing $\boldsymbol{y} = (\varphi _{1}, \varphi _{2}, \omega _{1}, \omega _{2})^{ \mathsf{T}}$, the matrices defining the boundary terms read

27 $$ \mathbf{L}:= \begin{pmatrix} \mathbf{I}_{2} & \mathbf{0}_{2\times 2} \end{pmatrix} , \quad \mathbf{U}:= \begin{pmatrix} \mathbf{0}_{2\times 2} & \mathbf{I}_{2} \end{pmatrix} , $$

where the matrices are composed of a horizontally concatenated 2×2 identity matrix $\mathbf{I}_{2}$ and a zero matrix $\mathbf{0}_{2\times 2}$. Imposing the right boundary terms ($x_{0}=0$), the residual vector and the shooting error function from Eq. (4) are given by:

28 $$ \boldsymbol{r}_{N} :=\boldsymbol{r}(\boldsymbol{y}_{N}, a) = \begin{pmatrix} \varphi _{1}(a) - \pi \\ \varphi _{2}(a) - \pi \\ \alpha \omega _{1}(a) \end{pmatrix} , \quad \mathrm{and} \quad \Gamma := \frac{1}{2}\boldsymbol{r}^{ \mathsf {T}}_{N} \boldsymbol{r}_{N}. $$

Herein, $\alpha =1\text{ s}$ is a weighting factor, which is set to 1 to keep the formulation simple. If we introduce the costate vector $\boldsymbol{\lambda} = (\lambda _{1}, \lambda _{2},\lambda _{3}, \lambda _{4})^{\mathsf{T}}$, the costate recursive scheme is obtained from Eq. (20). Owing to the length of the expressions, the costates in Eq. (20) and the gradient in Eq. (14) are not presented in their explicit forms here. For the numerical computations we choose the parameters: $l_{1}=l_{2}=0.5\text{ m}$, $s_{1}=s_{2}=0.25\text{ m}$, $m_{1}=m_{2}=1\text{ kg}$, $J_{1}=J_{2}=0.0208\text{ kg}\,\mathrm{m}^{2}$, $d_{1}=d_{2}=0.01\text{ Nms/rad}$ and $g=9.81\mathrm{m/s}^{2}$ and we provide an initial guess $\boldsymbol{u}=(\omega _{1}(0),\omega _{2}(0))=( 1~{\mathrm{rad}/s}, 1~{\mathrm{rad}/s})^{\mathsf{T}}$ and $a=0.1\text{ s}$.

#### Numerical results

**Fig. 2:**
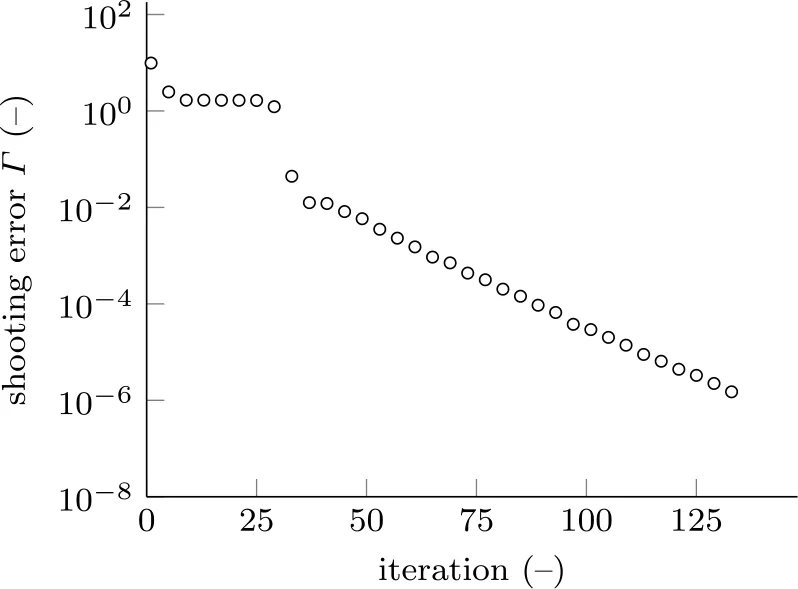
Convergence history of the double pendulum upswing

**Fig. 3:**
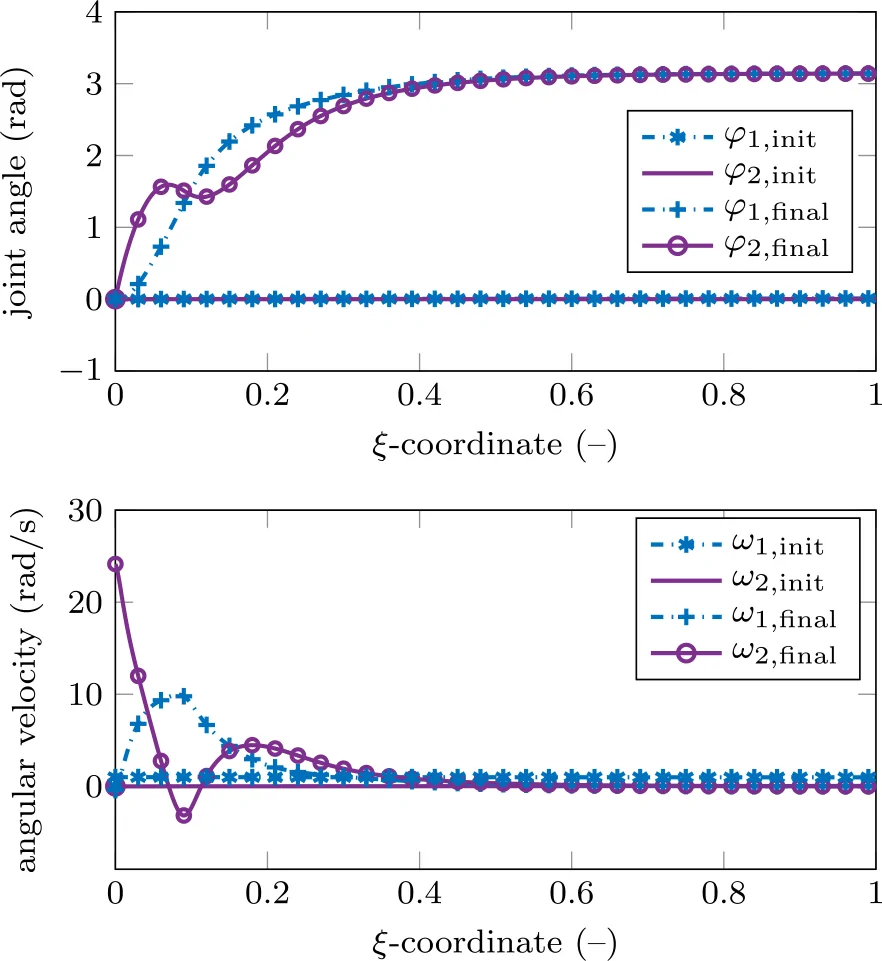
State histories of the double pendulum upswing

**Fig. 4:**
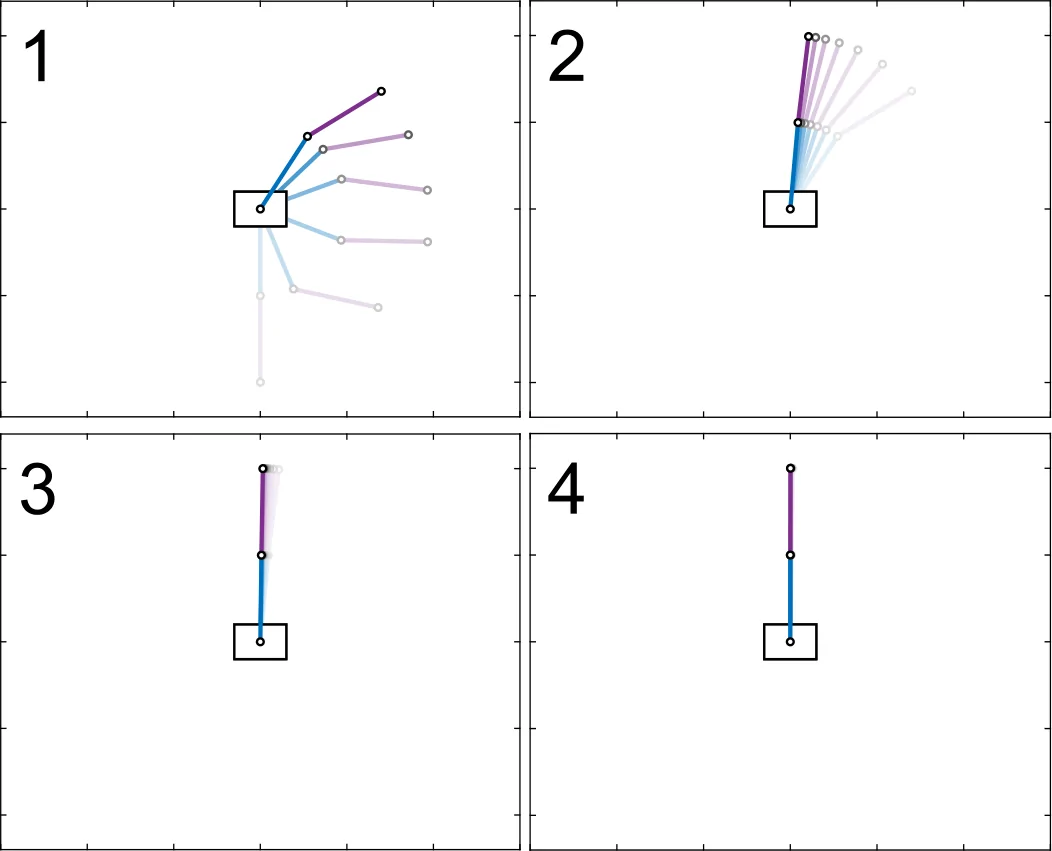
Trajectory sequence of the double pendulum upswing

The optimal initial condition set reads $\boldsymbol{u}^{*} = (\omega _{1}^{*}(0), \omega _{2}^{*}(0))^{ \mathsf{T}} = (2.8469~{\mathrm{rad}/\mathrm{s}}, 21.3620~{\mathrm{rad}/\mathrm{s}})^{ \mathsf{T}}$ and $a^{*} = 1.6219\text{ s}$, where $a^{*}$ corresponds to the identified time required for the pendulum to swing up to the upper equilibrium position. The results of the inverse double pendulum are summarized in Fig. 2–4. After approximately 130 iterations the final conditions are in good agreement with the desired boundary conditions. The convergence history of the optimization is shown in Fig. 2. The joint angles $\varphi _{1}$ and $\varphi _{2}$ and the angular velocities $\omega _{1}$ and $\omega _{2}$ are depicted in Fig. 3 and the motion sequence of the double pendulum swing up maneuver is illustrated in Fig. 4.

### Planar robot arm

**Fig. 5:**
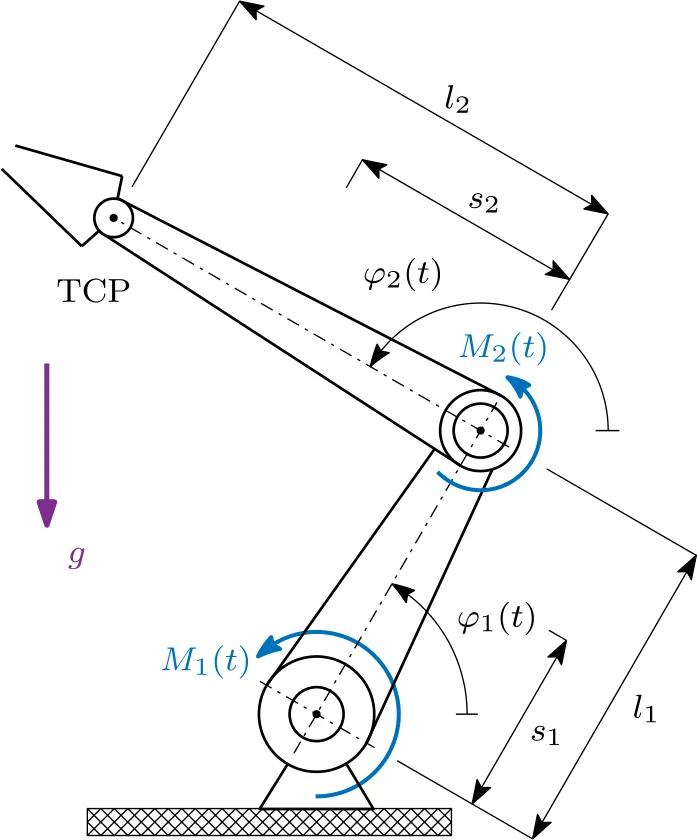
Geometric description of a planar two-axis robot

For the second example we consider a planar robot arm model depicted in Fig. 5. The robot consists of two revolute joints and two robot arms with the lengths $l_{1}$ and $l_{2}$. The robot is described by a minimal independent set of generalized coordinates $\varphi _{1}$ and $\varphi _{2}$. The two robot arms have the masses $m_{1}$ and $m_{2}$ and the moments of inertia $J_{1}$ and $J_{2}$ measured about their centers of gravity. Moreover, the robot is actuated by the drive torques $M_{1}$ and $M_{2}$ applied at its revolute joints and a mass $m_{3}$ is moved at the tool center point. Using the abbreviations:

29 $$ \begin{gathered} I_{a} = J_{1} + s_{1}^{2} m_{1} + l_{1}^{2} \left (m_{2} + m_{3} \right ) \quad I_{b} = J_{2} + s_{2}^{2} m_{2} + l_{2}^{2} m_{3} \\ I_{c} = l_{1} \left ( s_{2} m_{2} + l_{2} m_{3} \right ), \end{gathered} $$

and

30 $$ \begin{aligned} G_{a} = g \left [ s_{1} m_{1} + l_{1} \left (m_{2} + m_{3} \right ) \right ] \quad G_{b} = g \left (s_{2} m_{2} + l_{2} m_{3} \right ), \end{aligned} $$

the first-order state equations of the planar robot arm are given by

31 $$ \begin{aligned} \dot{\varphi}_{1} =& \, \omega _{1} \\ \dot{\varphi}_{2} =& \, \omega _{2} \\ \dot{\omega}_{1} =& \, \mu \Big[ I_{b} \big( M_{1} - M_{2} - G_{a} \cos (\varphi _{1})\big) - I_{c} \big( M_{2} - G_{b} \cos (\varphi _{2}) \big) \cos (\varphi _{1} - \varphi _{2}) \\ &-I_{c}\big(I_{b} \omega _{2}^{2} + I_{c} \omega _{1}^{2} \cos ( \varphi _{1}-\varphi _{2}) \big) \sin (\varphi _{1} - \varphi _{2}) \Big] \\ \dot{\omega}_{2} =& \, \mu \Big[I_{a} \big(M_{2} - G_{b} \cos ( \varphi _{2}) \big) + I_{c} \big(M_{2} - M_{1} + G_{a} \cos (\varphi _{1}) \big) \cos (\varphi _{1}-\varphi _{2}) \\ &+I_{c} \big( I_{a} \omega _{1}^{2} + I_{c} \omega _{2}^{2} \cos ( \varphi _{1}-\varphi _{2}) \big) \sin (\varphi _{1}-\varphi _{2}) \Big], \end{aligned} $$

in which we introduced the abbreviations $\mu := 1/\left (I_{a} I_{b} - I_{c}^{2} \cos ^{2}(\varphi _{1}- \varphi _{2})\right )$, $\omega _{1} := \dot{\varphi}_{1}$ and $\omega _{2} = \dot{\varphi}_{2}$. For an energy optimal manipulation, see [15, Sect. 5.5], we introduce the performance measure

32 $$ J = \frac{1}{2} \int _{0}^{T} \Big( M_{1}^{2} + M_{2}^{2} \Big) \mathrm{d}t, $$

which includes the squared signal energy of the robot to be minimized.

From the performance measure in Eq. (32) and the state equations in Eq. (31), the Hamiltonian reads

33 $$ \begin{aligned} \mathcal{H} =& \frac{1}{2} \left ( M_{1}^{2} + M_{2}^{2} \right ) + p_{1} \omega _{1} + p_{2} \omega _{2} \\ &+ p_{3} \mu \Big[ I_{b} \big( M_{1} - M_{2} - G_{a} \cos (\varphi _{1}) \big) - I_{c} \big( M_{2} - G_{b} \cos (\varphi _{2}) \big) \cos ( \varphi _{1} - \varphi _{2}) \\ &-I_{c}\big(I_{b} \omega _{2}^{2} + I_{c} \omega _{1}^{2} \cos ( \varphi _{1}-\varphi _{2}) \big) \sin (\varphi _{1} - \varphi _{2}) \Big] \\ &+ p_{4} \mu \Big[I_{a} \big(M_{2} - G_{b} \cos (\varphi _{2}) \big) + I_{c} \big(M_{2} - M_{1} + G_{a} \cos (\varphi _{1})\big) \cos ( \varphi _{1}-\varphi _{2}) \\ &+I_{c} \big( I_{a} \omega _{1}^{2} + I_{c} \omega _{2}^{2} \cos ( \varphi _{1}-\varphi _{2}) \big) \sin (\varphi _{1}-\varphi _{2}) \Big]. \end{aligned} $$

If we introduce $\boldsymbol{x} =(\varphi _{1}, \varphi _{2}, \omega _{1}, \omega _{2})^{\mathsf{T}}$ and $\boldsymbol{p} =(p_{1}, p_{2}, p_{3}, p_{4})^{\mathsf{T}}$, the adjoint equations are directly obtained from $\dot{\boldsymbol{p}} = -(\partial \mathcal{H}/ \partial \boldsymbol{x})^{\mathsf{T}}$ yielding:

34 $$ \begin{aligned} \dot{p}_{1} =&- \mu G_{a} I_{b} p_{3} \sin (\varphi _{1}) - \mu I_{c} \Big[ p_{3} \big( M_{2} - G_{b} \cos (\varphi _{2}) + I_{c} \omega _{1}^{2} \sin (\varphi _{1}-\varphi _{2}) \big) \\ &+ p_{4} \big( M_{1} - M_{2} - G_{a} \cos (\varphi _{1}) - I_{c} \omega _{2}^{2} \sin (\varphi _{1}-\varphi _{2}) \big) \Big] \sin ( \varphi _{1}-\varphi _{2}) \\ &+ \mu I_{c} \Big[ p_{3} \big( I_{b} \omega _{2}^{2} + I_{c} \omega _{1}^{2} \cos (\varphi _{1}-\varphi _{2}) \big) \\ &+p_{4} \big(G_{a} \sin (\varphi _{1}) - I_{a} \omega _{1}^{2} - I_{c} \omega _{2}^{2} \cos (\varphi _{1}-\varphi _{2}) \big) \Big] \cos ( \varphi _{1}-\varphi _{2}) \\ \dot{p}_{2} =& - \mu G_{b} I_{a} p_{4} \sin (\varphi _{2}) - \mu I_{c} \Big[ p_{3} \big( G_{b} \cos (\varphi _{2}) - M_{2} - I_{c} \omega _{1}^{2} \sin (\varphi _{1}-\varphi _{2}) \big) \\ &+p_{4} \big(M_{2} - M_{1} + G_{a} \cos (\varphi _{1}) + I_{c} \omega _{2}^{2} \sin (\varphi _{1}-\varphi _{2})\big) \Big] \sin ( \varphi _{1}-\varphi _{2}) \\ &+\mu I_{c} \Big[p_{3}\big(G_{b} \sin (\varphi _{2}) + I_{b} \omega _{2}^{2} + I_{c} \omega _{1}^{2} \cos (\varphi _{1}-\varphi _{2}) \big) \\ &-p_{4} \big(I_{a} \omega _{1}^{2} + I_{c} \omega _{2}^{2} \cos ( \varphi _{1} -\varphi _{2})\big) \Big] \cos (\varphi _{1} -\varphi _{2}) \\ \dot{p}_{3} =& - p_{1} + 2 \mu I_{c} \omega _{1} \big(I_{c} p_{3} \cos (\varphi _{1}-\varphi _{2}) - I_{a} p_{4} \big) \sin (\varphi _{1}- \varphi _{2}) \\ \dot{p}_{4} =& - p_{2} + 2 \mu I_{c} \omega _{2} \big(I_{b} p_{3} - I_{c} p_{4} \cos (\varphi _{1}- \varphi _{2}) \big) \sin (\varphi _{1}- \varphi _{2}). \end{aligned} $$

From the algebraic relationship $\boldsymbol{0} = (\partial \mathcal{H}/\partial \boldsymbol{M})^{ \mathsf{T}}$, we can solve for the drive signals $\boldsymbol{M} =(M_{1}, M_{2})^{\mathsf{T}}$, which yields

35 $$ \begin{aligned} M_{1} &= \mu \Big(I_{c} \cos (\varphi _{1}-\varphi _{2}) p_{4} - I_{b} p_{3}\Big), \\ M_{2} &= \mu \Big[\left (I_{b} + I_{c} \cos (\varphi _{1}-\varphi _{2}) \right ) p_{3} - \left (I_{a} + I_{c} \cos (\varphi _{1}-\varphi _{2}) \right ) p_{4}\Big]. \end{aligned} $$

Substituting Eq. (35) in the state equations in Eq. (31) and the adjoint equations in Eq. (34), $M_{1}$ and $M_{2}$ can be eliminated. Before the boundary value problem can be posed, the boundary conditions must be specified for the time interval $t \in \left [x_{0},x_{f}\right ] = \left [0, T\right ]$. The left boundary conditions of the robot are given by:

36 $$ \varphi _{1}(0) = -\pi /4{\text{ rad}} \quad \varphi _{2}(0) = 0{\text{ rad}} \quad \omega _{1}(0) = 0~{\mathrm{rad}/s} \quad \omega _{2}(0) = 0~{\mathrm{rad}/s}. $$

Moreover, the right boundary conditions are given by

37 $$ \varphi _{1}(T) = \pi /2{\text{ rad}} \quad \varphi _{2}(T) = \pi /2{\text{ rad}} \quad \omega _{1}(T) = 0~{\mathrm{rad}/s} \quad \omega _{2}(T) = 0~{\mathrm{rad}/s}. $$

#### Solution strategy

The state equations can be readily written in the form of Eq. (2) by collecting the states of Eq. (31) and the adjoint variables of Eq. (34) in a vector $\boldsymbol{y} = (\varphi _{1}, \varphi _{2}, \omega _{1}, \omega _{2}, p_{1}, p_{2}, p_{3}, p_{4})^{\mathsf{T}}$. Hence, the matrices defining the boundary terms are given by

38 $$ \mathbf{L}:= \begin{pmatrix} \mathbf{I}_{4} & \mathbf{0}_{4\times 4} \end{pmatrix} , \quad \mathbf{U}:= \begin{pmatrix} \mathbf{0}_{4\times 4} & \mathbf{I}_{4} \end{pmatrix} , $$

where, analogously to the double pendulum example above, the matrices consist of the horizontally concatenated 4×4 identity matrix $\mathbf{I}_{4}$ and the 4×4 zero matrix $\mathbf{0}_{4\times 4}$. For $x_{0}=0$ the right-hand side boundary terms and the shooting error function are defined by

39 $$ \boldsymbol{r}_{N} :=\boldsymbol{r}(\boldsymbol{y}_{N}, a) = \begin{pmatrix} \varphi _{1}(a) - \pi /2 \\ \varphi _{2}(a) - \pi /2 \\ \omega _{1}(a) \\ \omega _{2}(a) \end{pmatrix} , \quad \mathrm{and} \quad \Gamma := \frac{1}{2}\boldsymbol{r}^{ \mathsf {T}}_{N} \boldsymbol{r}_{N} + \frac{\eta}{2}\left (a-T\right )^{2}, $$

where $\alpha _{1}=1\text{ s}$, $\alpha _{2}=1\text{ s}$ and $\eta =1~{1/s^{2}}$ are weighting factors, which could be used to regulate the impact on the shooting error. Introducing the costate vector $\boldsymbol{\lambda} = (\lambda _{1}, \lambda _{2},\dots ,\lambda _{8})^{ \mathsf{T}}$, the associated costate equations follow directly from Eq. (20) and the gradient formula is given by Eq. (14). Due to the length of these expressions, we refrain from presenting the costate equations and the gradient formula here. For the numerical computations we consider the final time $T = 1\text{ s}$ fixed and we choose the parameter set $l_{1}=l_{2}=1\text{ m}$, $s_{1}=s_{2}=0.5\text{ m}$, $m_{1}=m_{3}=1\text{ kg}$, $m_{2} = 0.5\text{ kg}$, $J_{1} = 0.0833\text{ kg}\,\mathrm{m}^{2}$, $J_{2} =0.0417\text{ kg}\,\mathrm{m}^{2}$ and $g = 9.81\text{ m/s}^{2}$. The initial conditions $\varphi _{1}(0)=-\pi /4{\text{ rad}}$, $\varphi _{2}(0)=0{\text{ rad}}$ and $\omega _{1}(0)=\omega _{2}(0)= 0~{\mathrm{rad}/s}$ are prescribed, while we provide a guess for the unknown conditions by $\boldsymbol{u} =(p_{1}(0),p_{2}(0),p_{3}(0),p_{4}(0))^{\mathsf{T}} = (0~\text{ N}^{2}\mathrm{m}^{2}\text{ s/rad}, 0\text{ N}^{2}\,\mathrm{m}^{2}\text{ s/rad},0\text{ N} \, \mathrm{m}^{3} \, \mathrm{kg}, 0\text{ N} \, \mathrm{m}^{3} \, \mathrm{kg})^{\mathsf{T}}$ and $a= 0.1\text{ s}$.

#### Numerical results

**Fig. 6:**
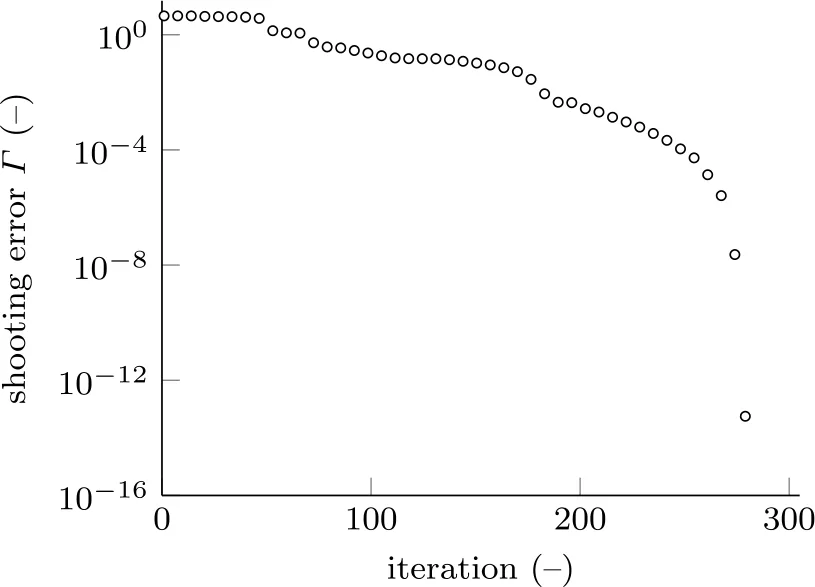
Convergence history of the energy optimal point-to-point maneuver

**Fig. 7:**
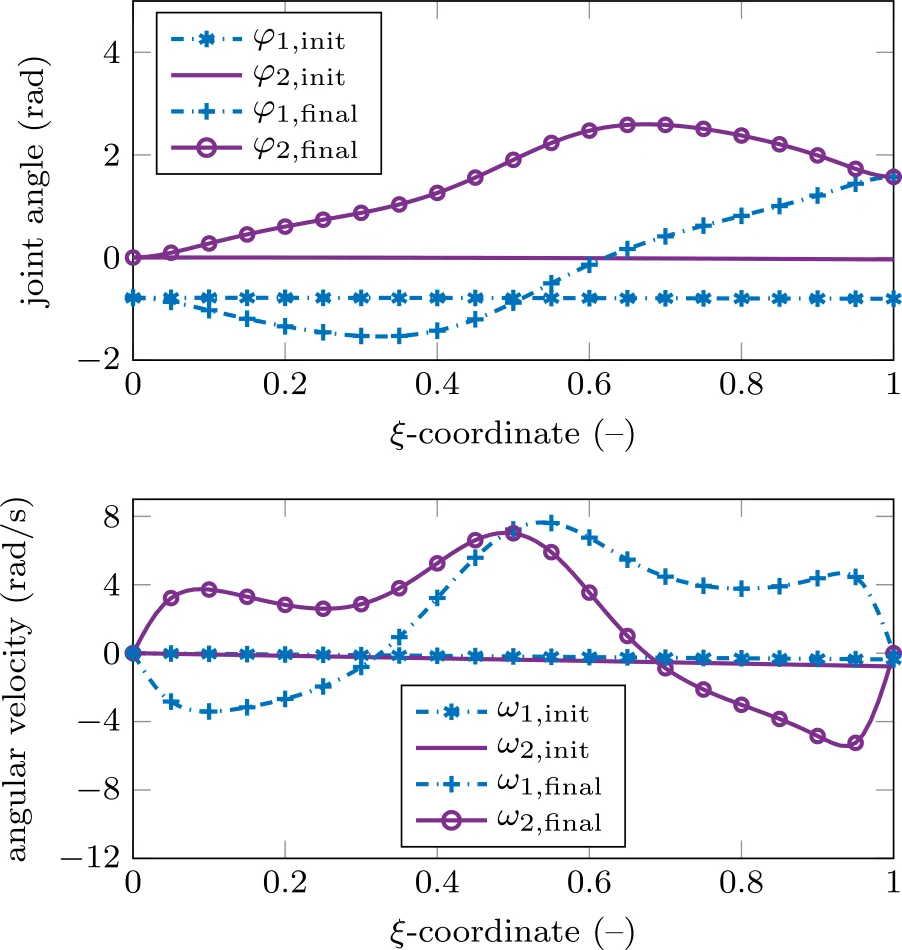
State histories of the energy optimal point-to-point maneuver

**Fig. 8:**
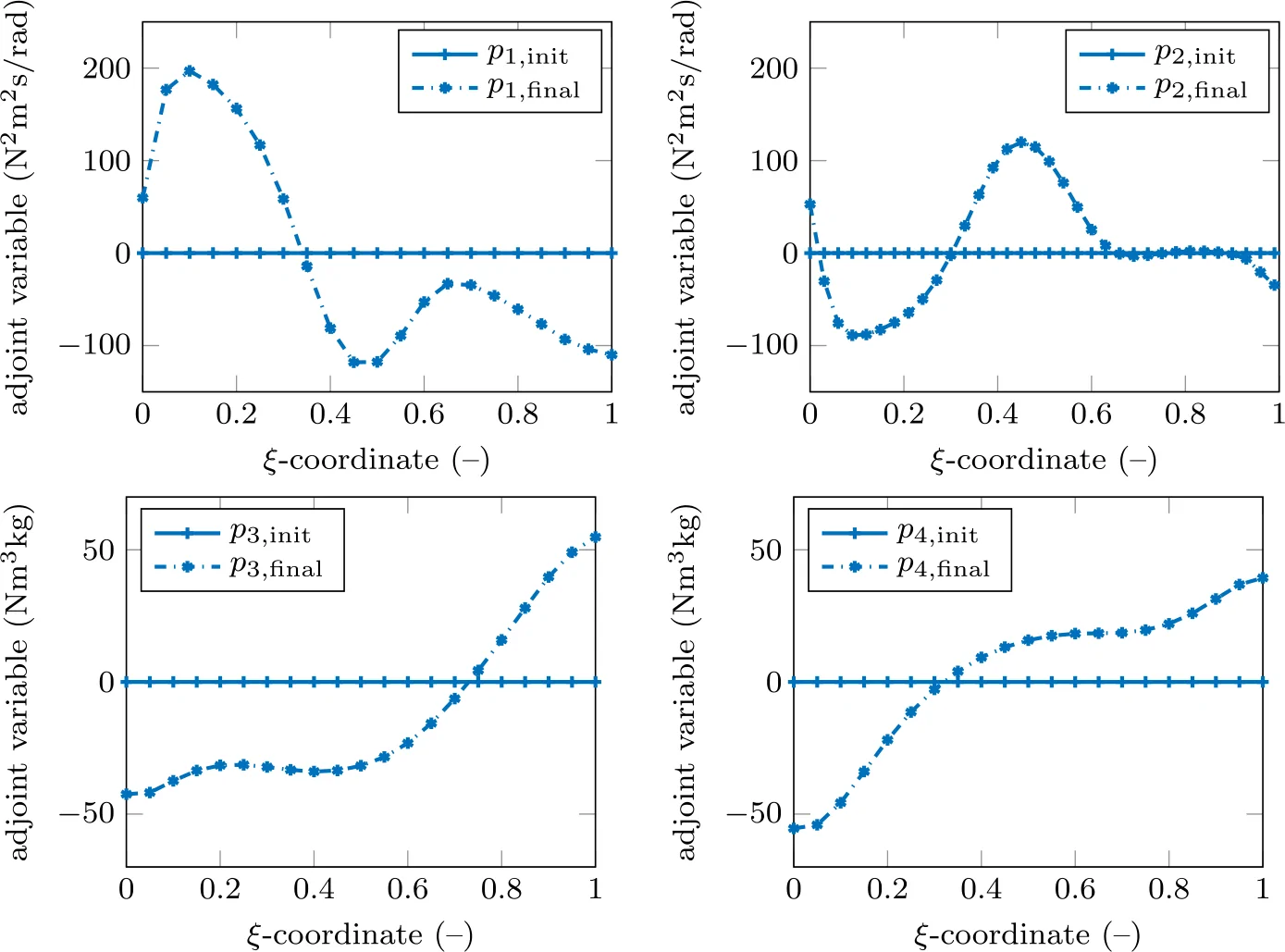
Adjoint variables of the energy optimal point-to-point maneuver

**Fig. 9:**
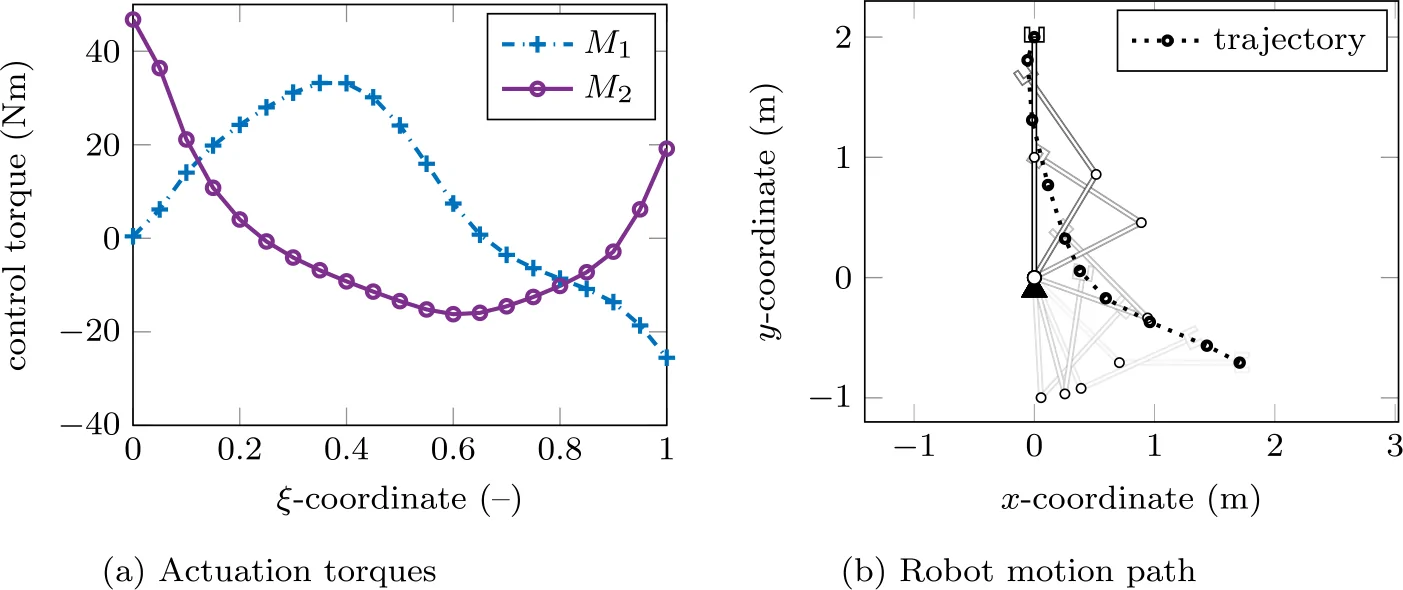
Optimal control solution for a two-arm robot

The results of the underlying TPBVP for the robot control problem are summarized in Figs. 6–9. The numerical solution of the differential equations was obtained using a Runge-Kutta discretization with a time step size of 0.01 and a solver error tolerance of $10^{-6}$. By using a quasi-Newton method like the BFGS algorithm, the solution can be found within 280 iterations. The convergence history, i.e., the RMS error of the optimization process, is illustrated in Fig. 6. The identified initial conditions are given by $\boldsymbol{u}^{*} = (p_{1}^{*}(0), p_{2}^{*}(0),p_{3}^{*}(0),p_{4}^{*}(0))^{ \mathsf{T}} = ( 59.7466\text{ N}^{2}\mathrm{m}^{2} \mathrm{s/rad}, 52.7712\text{ N}^{2}\mathrm{m}^{2} \mathrm{s/rad}, -42.4782\text{ N} \, \mathrm{m}^{3} \, \mathrm{kg}, -55.4294\text{ N} \, \mathrm{m}^{3} \, \mathrm{kg})^{\mathsf{T}}$ and $a^{*} = 1\text{ s}$, where $a^{*}=T$ corresponds to the identified time required for the energy optimal point-to-point maneuver. Moreover, the initial cost residual, i.e., the difference between the computed and target right boundary conditions, quantifies the error $\Gamma (\boldsymbol{u},a) = 4.888$. The final shooting error is smaller than $\Gamma (\boldsymbol{u}^{*},a^{*}) = 4.54 \times 10^{-15}$ and the state trajectories of $\varphi _{1}$, $\varphi _{2}$, $\omega _{1}$ and $\omega _{2}$ over time are shown in Fig. 7. Figure 8 illustrates the adjoint variables $p_{1}$, $p_{2}$, $p_{3}$ and $p_{4}$ derived from the underlying optimal control problem. Both the initial guess and the resulting optimal solution are shown, illustrating the sensitivity of the cost functional to variations in the state trajectory. Moreover, the energy-optimal control torques, as well as the resulting trajectory of the two-arm robot, are shown in Fig. 9a and Fig. 9b. Note that the control torques are computed by Eq. (35) in a post-processing step after the optimization.

### Restricted three-body problem

As a final example, we consider a boundary value problem for the computation of periodic orbits in the restricted three-body problem. In the restricted three-body problem [1], the motion of a small mass is considered, e.g., a satellite, under the effect of two celestial bodies moving in circular orbits around their common center of mass. We consider spatial trajectories of a satellite in a rotating coordinate frame where its x-axis is aligned along the celestial bodies with the masses $M_{1}$ and $M_{2}$, its y-axis is in the plane of their orbits and its origin is located at the center of mass. Moreover, we choose the length, time and mass scale such that the distance between the primaries, their mean angular velocity, and the sum of their masses are unity. Then, the restricted three-body problem (cf. [1]) can be described by the first-order differential equations

40 $$\begin{aligned} \dot{x} &= u \\ \dot{y} &= v \\ \dot{z} &= w \\ \dot{u} &= 2v + x + \frac{\mu \left (1-\mu -x\right )}{\sqrt{\left ( \left (1-\mu -x\right )^{2} +y^{2} +z^{2} \right )^{3}}} - \frac{\left (1-\mu \right ) \left (\mu +x\right )}{\sqrt{\left (\left (\mu +x\right )^{2} +y^{2} +z^{2}\right )^{3}}} \\ \dot{v} &= -2 u + y - \frac{\mu y}{\sqrt{\left (\left (1-\mu -x\right )^{2}+y^{2}+z^{2}\right )^{3}}} - \frac{y\left (1-\mu \right )}{\sqrt{\left (\left (\mu +x\right )^{2} + y^{2} + z^{2}\right )^{3}}} \\ \dot{w} &= - \frac{\mu z}{\sqrt{\left (\left (1-\mu -x\right )^{2}+y^{2}+z^{2}\right )^{3}}} - \frac{z \left (1-\mu \right )}{\sqrt{\left (\left (\mu +x\right )^{2} + y^{2} +z^{2}\right )^{3}}}, \end{aligned}$$

where x, y and z denote the coordinates of the infinitesimal mass (satellite) in the rotating frame, and u, v and w denote the associated velocity components in terms of this length and time scales. Moreover, the parameter 0<μ<1 denotes the mass ratio $\mu = M_{2}/(M_{1} + M_{2})$. In the following computations, we adopt μ=0.25, which does not correspond to any configuration in the Solar System. We consider two different sets of boundary conditions, which result in periodic orbits around the first and the second Lagrange point. Notice that there are five stationary solutions (equilibrium positions) of the restricted three-body problem which all lie in the x-y-plane and are referred to as Lagrange points, cf. [1]

We consider two different sets of boundary conditions, which result in different trajectories: the first leads to an orbit around the first Lagrange point (L1), while the second case results in an orbit around the second Lagrange point (L2). In the following computations, we adopt μ=0.25, which does not correspond to any configuration in the Solar System. The equilibrium positions lie in the x-y-plane and are five stationary points of the Hill surface, which are referred to as Lagrange points.

*CASE I: A periodic orbit around L1*: For $t_{0}=0$ the initial conditions (left-hand side) read

41 $$ \begin{gathered} x(0) = 0.284029, \quad y(0) = 0, \quad z(0) = \mathrm{free}, \\ u(0) = 0, \quad v(0) = \mathrm{free}, \quad w(0) = 0 \end{gathered} $$

The period duration T is considered free. Hence, the right boundary conditions ($x_{f} = T$) are prescribed by

42 $$ \begin{gathered} x(T) = \mathrm{free}, \quad y(T) = 0, \quad z(T) = \mathrm{free}, \\ u(T) = 0, \quad v(T) = \mathrm{free}, \quad w(T) = 0, \quad T = \mathrm{free}. \end{gathered} $$

*CASE II: A periodic orbit around L2*: Here, only the initial x-coordinate of the satellite from case I is varied, leading to a distinct orbit around the Lagrange point L2. For $t_{0}=0$ the initial conditions now read

43 $$ \begin{gathered} x(0) = 1.056, \quad y(0) = 0, \quad z(0) = \mathrm{free}, \\ u(0) = 0, \quad v(0) = \mathrm{free}, \quad w(0) = 0 \end{gathered} $$

and the boundary conditions on the right-hand side ($x_{f} = T$) are the same as in Eq. (42).

#### Solution strategy

Due to the free period duration T, a total of seven boundary conditions are imposed by Eq. (41) and Eq. (42) or, respectively, by Eq. (43). By introducing $\boldsymbol{y} = (x, y, z, u, v, w)^{\mathsf{T}}$, the matrices defining the boundary conditions for both cases I and II are as follows:

44 $$ \mathbf{L}:= \begin{pmatrix} 1 & 0 & 0 & 0 & 0 & 0 \\ 0 & 1 & 0 & 0 & 0 & 0 \\ 0 & 0 & 0 & 0 & 0 & 0 \\ 0 & 0 & 0 & 1 & 0 & 0 \\ 0 & 0 & 0 & 0 & 0 & 0 \\ 0 & 0 & 0 & 0 & 0 & 1 \end{pmatrix}, \quad \mathbf{U}:= \begin{pmatrix} 0 & 0 & 0 & 0 & 0 & 0 \\ 0 & 0 & 0 & 0 & 0 & 0 \\ 0 & 0 & 1 & 0 & 0 & 0 \\ 0 & 0 & 0 & 0 & 0 & 0 \\ 0 & 0 & 0 & 0 & 1 & 0 \\ 0 & 0 & 0 & 0 & 0 & 0 \end{pmatrix}. $$

Imposing the right boundary terms ($x_{0}=0$), the residual vector and the shooting error function from Eq. (4) are given by:

45 $$ \boldsymbol{r}_{N} :=\boldsymbol{r}(\boldsymbol{y}_{N}, a) = \begin{pmatrix} y(a) \\ \alpha u(a) \\ \alpha w(a) \end{pmatrix} , \quad \mathrm{and} \quad \Gamma := \frac{1}{2}\boldsymbol{r}^{ \mathsf {T}}_{N} \boldsymbol{r}_{N}. $$

Herein, $\alpha =1\text{ s}$ is again a weighting factor. Furthermore, the costate vector is given by $\boldsymbol{\lambda} = (\lambda _{1}, \lambda _{2},\lambda _{3}, \lambda _{4},\lambda _{5},\lambda _{6})^{\mathsf{T}}$ and the associated recursive scheme is obtained from Eq. (20). As in previous examples, the costate equations and the update formula in Eq. (14) are not presented here due to their length. For the boundary conditions defined in the first case, Eq. (41) and Eq. (42), we use the initial guess $\boldsymbol{u}=(z(0),v(0))=(0,1)^{\mathsf{T}}$ and $a=1\text{ s}$. For the second case, Eq. (43) and Eq. (42) apply and we make the initial guess $\boldsymbol{u}=(z(0),v(0))=(0,1)^{\mathsf{T}}$ and $a=4\text{ s}$.

#### Numerical results

For the first case, the optimal initial condition set reads $\boldsymbol{u}^{*} = (z^{*}(0), v^{*}(0))^{\mathsf{T}} = (0, 0.832162)^{\mathsf{T}}$ and $a^{*} = 2.82532$, where $a^{*}$ corresponds to the identified period duration time.

**Fig. 10:**
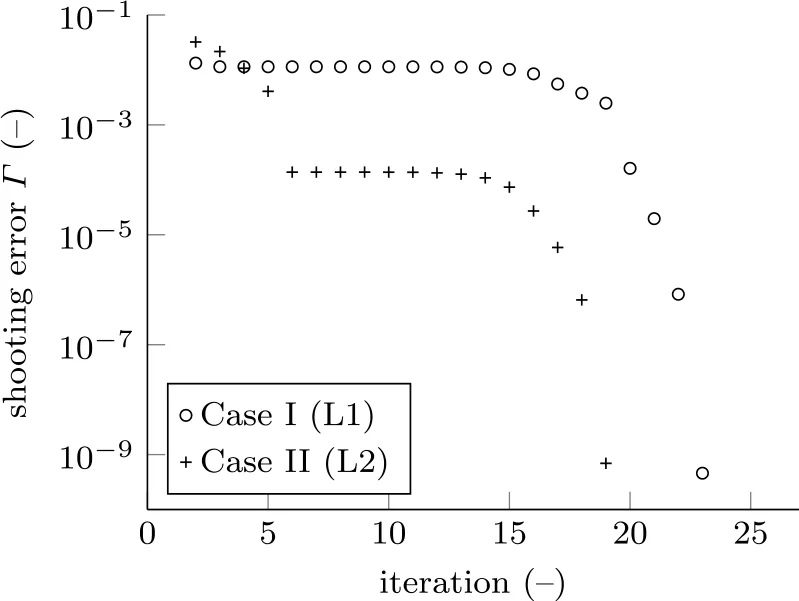
Convergence history of the restricted three-body problem

**Fig. 11:**
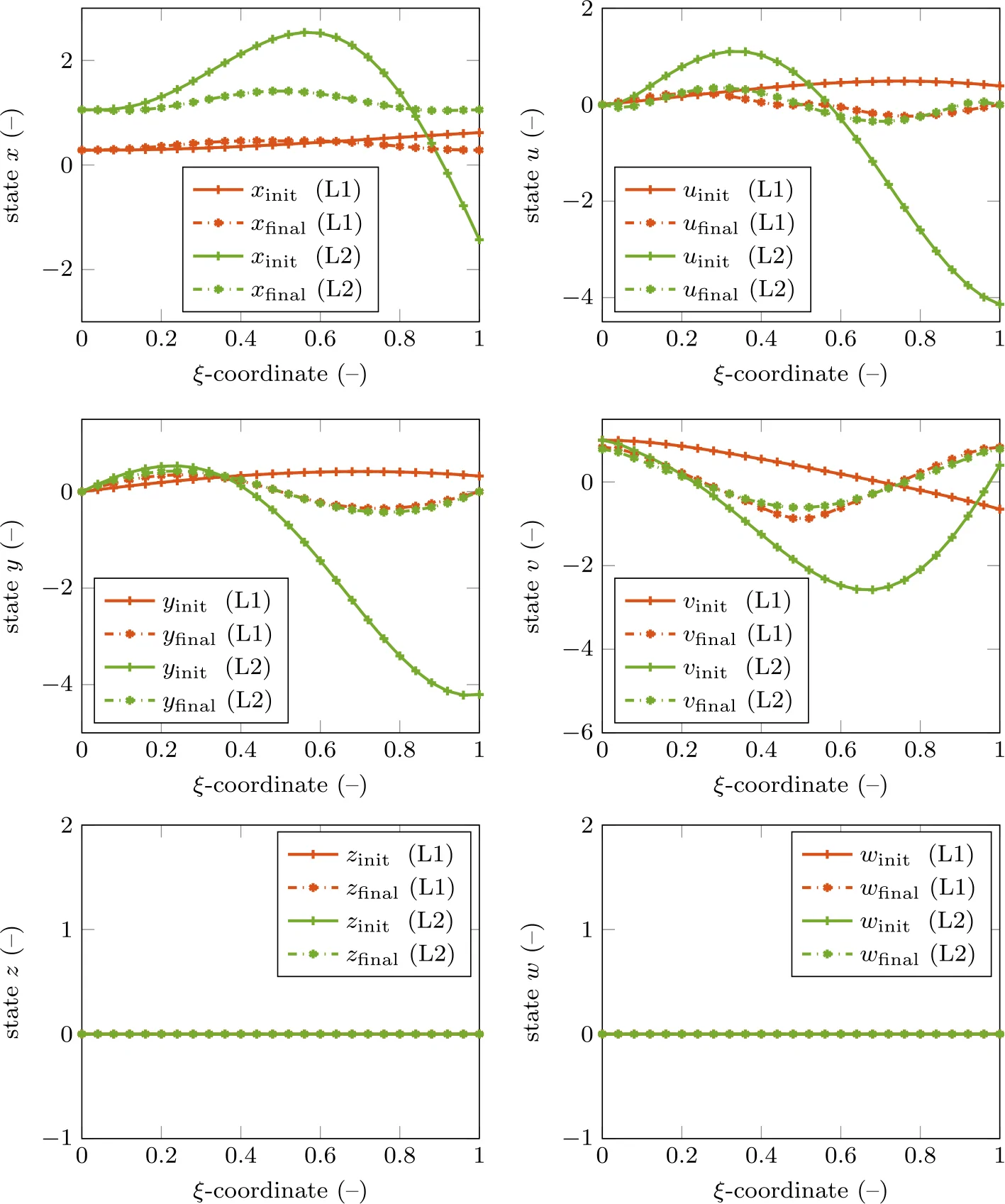
State histories of the restricted three-body problem (case I and II). Note that the distance between the celestial bodies defines one length unit

**Fig. 12:**
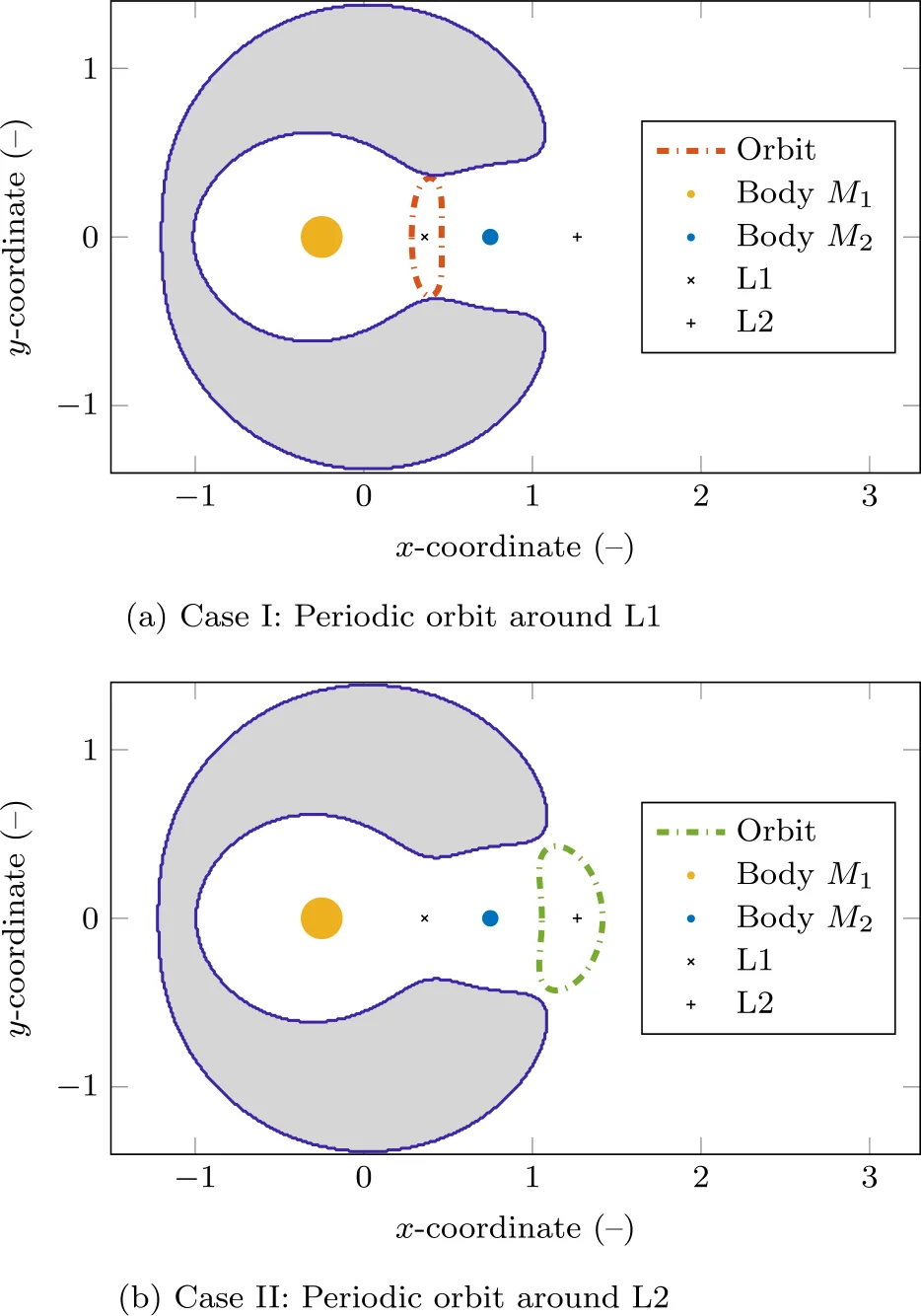
Periodic orbits of the restricted three-body problem. As before, the distance between the celestial bodies serves as the unit length

For the second case, the optimal condition set reads $\boldsymbol{u}^{*} = (z^{*}(0), v^{*}(0))^{\mathsf{T}} = (0, 0.784383)^{\mathsf{T}}$ and the period duration time $a^{*} = 4.23361$. Figures 10–12 provide a summary of the results obtained for the restricted three-body problem. After 23 iterations (case I) and 19 iterations (case II) the right boundary conditions are in good agreement with the desired boundary conditions. The convergence history of the optimization is shown in Fig. 10. The coordinates x, y, z and the associated velocities u, v and w are depicted in Fig. 11 for both Case I and Case II. Moreover, the motion of the periodic orbits are illustrated in Fig. 12a and in Fig. 12b. The contour lines on the gray-shaded Hill surface (μ=0.25) show the accessible regions to a probe under the energy constraint, i.e., those bounded by the Hill surface.

## Conclusion

We have introduced a modification of the classical shooting method for solving TPBVPs. From the numerical results for the double pendulum and the robot model, the DSM shows robust behavior and delivers accurate solutions, even under difficult dynamic conditions and with sensitive initial conditions. In summary, we provide a brief overview of our experience with the DSM so far, highlighting its advantages and disadvantages of the proposed method over traditional approaches: (i) The initial values are specified only at the beginning of the considered interval, rather than at several intermediate points as is the case with the multiple shooting method. Hence, the number of values that need to be considered is reduced. (ii) Moreover, the transformation of the differential equations to a unit interval achieves a similar effect as in multiple shooting method, as the solution does not have unstable modes in small intervals. (iii) The method provides an exact gradient computation via the costate variable approach, avoiding numerical differentiation and its associated disturbance parameter selection challenges. (iv) However, it should be mentioned that in some scenarios the solution may converge to a local minimum where the boundary conditions are not met. The convergence to a global or local minimum in the optimization process is influenced by the problem setup and the initial guess. In the case of a local minimum, one can either try alternative starting values or initially reduce the computational interval by choosing a smaller transformation parameter.

Overall, the DSM requires low implementation effort while providing a robust approach for solving TPBVPs in multibody dynamics. In a future work considering more complex problems, the approach can be extended to differential-algebraic equation (DAE) systems, which arise often in multibody dynamics due to kinematic constraints and closed-loop mechanisms. Adapting the DSM to handle DAEs would involve developing strategies to maintain both the algebraic constraints and the stability of the integration.

## Data Availability

No datasets were generated or analysed during the current study.
